# Acetyl Coenzyme A Synthase 2 Acts as a Prognostic Biomarker Associated with Immune Infiltration in Cervical Squamous Cell Carcinoma

**DOI:** 10.3390/cancers13133125

**Published:** 2021-06-22

**Authors:** Chia-Jung Li, Yi-Han Chiu, Chung Chang, Yuan-Chin Ivan Chang, Jim Jinn-Chyuan Sheu, An-Jen Chiang

**Affiliations:** 1 Department of Obstetrics and Gynecology, Kaohsiung Veterans General Hospital, Kaohsiung 813, Taiwan; cjli@vghks.gov.tw; 2 Institute of BioPharmaceutical Sciences, National Sun Yat-sen University, Kaohsiung 804, Taiwan; 3 Department of Microbiology, Soochow University, Taipei 111, Taiwan; chiuyiham@scu.edu.tw; 4 Department of Applied Mathematics, National Sun Yat-sen University, Kaohsiung 804, Taiwan; cchang@math.nsysu.edu.tw; 5 Institute of Statistical Science, Academia Sinica, Taipei 115, Taiwan; ycchang@sinica.edu.tw; 6 Institute of Biomedical Sciences, National Sun Yat-sen University, Kaohsiung 804, Taiwan; jimsheu@mail.nsysu.edu.tw

**Keywords:** cervical cancer, ACSS2, prognosis, immune

## Abstract

**Simple Summary:**

Cellular metabolism has become a key determinant of cancer cell and immune cell viability and function. To sustain the enormous anabolic demands, tumors adopt a specialized metabolism different from that of normal cells. Tumor cells synthesize acetyl-CoA by uptake of extracellular acetic acid via acetyl coenzyme A synthetase 2 (ACSS2) to provide a carbon source for tumor cells. We found that the expression level of ACSS2 was significantly higher in CESC patients than in normal cells, and confirmed a positive correlation between the level of immune infiltration and ACSS2, thus ACSS2 as a key enzyme of tumor energy metabolism has become a new focus for researchers.

**Abstract:**

Cervical squamous cell carcinoma (CESC) is one of the most common malignant tumors in women worldwide with a low survival rate. Acetyl coenzyme A synthase 2 (ACSS2) is a conserved nucleosidase that converts acetate to acetyl-CoA for energy production. Our research intended to identify the correlations of ACSS2 with clinical prognosis and tumor immune infiltration in CESC. ACSS2 is highly expressed in many tumors and is involved in the progression and metastasis of these tumors. However, it is not clear how ACSS2 affects CESC progression and immune infiltration. Analysis of the cBioPortal, GEPIA2, UALCAN, and TCGA databases showed that ACSS2 transcript levels were significantly upregulated in multiple cancer types including CESC. Quantitative RT-PCR analysis confirmed that ACSS2 expression was significantly upregulated in human cervical cancer cells. Here, we performed tissue microarray analysis of paraffin-embedded tissues from 240 cervical cancer patients recorded at FIGO/TNM cancer staging. The results showed that ACSS2 and PDL1 were highly expressed in human CESC tissues, and its expression was associated with the clinical characteristics of CESC patients. TIMER database analysis showed that ACSS2 expression in CESC was associated with tumor infiltration of B cells, CD4+ and CD8+ T cells, and cancer-associated fibroblasts (CAF). Kaplan–Meier survival curve analysis showed that CESC with high ACSS2 expression was associated with shorter overall survival. Collectively, our findings establish ACSS2 as a potential diagnostic and prognostic biomarker for CESC.

## 1. Introduction

Cervical cancer is one of the most common malignancies in female patients, and despite the improved prognosis through early detection, cervical cancer remains the second most common cause of death in women [1]. Although more than 90% of patients with early-stage Cervical cancer are curable, the prognosis of patients with advanced cervical cancer remains poor, especially for those with metastatic Cervical cancer [2,3]. Therefore, new potential targets need to be identified for cervical cancer treatment.

ACSS2 belongs to the acetyl coenzyme A synthase short chain enzyme family, enzymes that convert acetate to acetyl coenzyme A, an important intermediate metabolite [4,5,6]. The three known isoforms of acyl-CoA short chain synthase are expressed in humans encoded by the *ACSS1*, *ACSS2*, and *ACSS3* genes. While ACSS1 and ACSS3 of the ACSS family are located in the mitochondria, ACSS2 is located in the cytoplasm and nucleus [7,8,9]. Since cancer cells use acetate as a carbon source, ACSS2 is critical for tumor metabolism in a hypoxic and glucose-restricted environment, leading to a shift in metabolism from aerobic glycolysis to oxidative phosphorylation (OXPHOS) [8,10]. ACSS2 controls the contribution of acetate to fatty acid synthesis and supports the biosynthesis of membrane phospholipids in breast cancer [10]. It helps cancer cells to survive in a hypoxic environment through lipogenesis [8]. It also promotes lipid synthesis and transcription of cell proliferation genes in breast and hepatocellular carcinomas [11,12]. ACSS2 promotes OXPHOS and lipid synthesis in NSCLC and ESCC cells, leading to a metabolic reprogramming and enhanced invasion [13,14]. The field of immunometabolism is a research area that studies the effects of metabolism on the immune response and has attracted great interest in recent years [15,16]. Previously, it was reported that the metabolism of immune cells undergoes dramatic changes during activation, for example, in response to antigen recognition in T cells or pathogen sensing in macrophages, which in turn increases cellular glucose utilization [17]. Therefore, we attempted to dissect the possible regulatory mechanisms of immune metabolism in CESC through the present study.

To understand the mechanism of ACSS2 and immune cell crosstalk in CESC, we analyzed the CESC cohort from the TCGA dataset using a diverse database. Collected genes were identified by evaluating the database data and comparing protein–protein interaction (PPI) networks. Finally, to validate the database results, we not only analyzed ACSS2 but also further analyzed programmed death ligand 1 (PD-L1) expression and verified the association between ACSS2 gene expression and immune cell infiltration.

## 2. Materials and Methods

### 2.1. Ethic Statement

The retrospective study was approved by the Institutional Review Board of Kaohsiung Veterans General Hospital (VGHKS15-CT6-09) and conformed to the current ethical principles of the Declaration of Helsinki.

### 2.2. Human Tissue Microarray (TMA) Immunohistochemical Analysis

The study randomly collected 240 CESC patient specimens for TMA, with exclusion criteria of congenital disease and clinical data loss. Patients’ prognosis and survival will be tracked through the hospital cancer registry system. Regular follow-ups will be performed every three months for the first three years, with annual CT exams during that time. Patients surviving more than three years will be followed up every six months or excluded if untraceable. The percentage of patients with biopsy type was 5% incisional biopsy, 6% conization, and 89% hysterectomy. HE stained slides from 240 CESC patients had to be reviewed by two pathologists to determine the area of interest to be cored and to establish the cohort of patients with acceptable amounts of target tissue for the specified TMA design. Tumor tissues from formalin-fixed, paraffin embedded tissue blocks of carcinomas with a core size of 1.5 mm were assessed. Sampling sites including 2 tumor sites and 1 non-tumor site were marked on each donor block by a pathology physician, and the tissue cylinders precisely arrayed into recipient blocks each with a core size of 1.5 mm. The blocks of embedded tissue for TMA were performed using a manual TMA (Beecher Instruments, Silver Spring, MD, USA); and the recipient was incubated overnight at 37 °C before sectioning. Immunohistochemical analysis in the TMA sections was carried out as described in a previous study [17]. The TMA sections (5 μm) were deparaffinized and incubated with 10 μg/mL proteinase K (Sigma-Aldrich, St. Louis, MO, USA) at 37 °C for 30 min. Quality assurance tests and the confirmation of diagnoses were carried out by staining the TMA sections with hematoxylin and eosin (HE). The slides were treated with anti-ACSS2 antibody (1:200, A6472, ABclonal, MA, USA), anti-PDL1 (1:200, A19135, ABclonal). All the slides were investigated under a microscope (BX50, OLYMPUS, Tokyo, Japan) and evaluated by two pathological physicians and the digital pathological biopsy scanning services from Biotechnology Corporation. The characteristics of all the patients included in this study are listed in Table 1.

### 2.3. Evaluation of the Human TMA Sections

We evaluated and scored TMA sections (ACSS2 and PDL1) and scored the signal of IHC positively stained tumor tissue as the percentage of labeled cells (0–100%); and intensity score (negative: −; weak: +, moderate: ++ or strong: +++) and recorded the percentage of IHC staining signal and the staining intensity score for histological score (H score, range 0–300). The H-score defines the percentage of cells with major staining intensity and staining intensity levels and assigns the H-score with the formula: [1 × (% cells 1+) + 2 × (% cells 2+) + 3 × (% cells 3+)]. For further statistical analysis, the tumors with H scores of 0–100, 101–200, and 201–300 were classified.

### 2.4. Cells and Cell Culture

Cervical cancer cell lines HeLa cells (BCRC#60005, Hsinchu, Taiwan) and CC7T/VGH cells (BCRC#60195), and CaSki cells (ATCC#CRL-1550) were used and cultured in DMEM supplemented with 10% fetal bovine serum (ThemoFisher Scientific, Waltham, MA, USA) in a humidified atmosphere of 95% air and 5% CO_2_ at 37 °C.

### 2.5. RNA Extraction and Real-Time PCR

RNA was extracted using the EasyPrep Total RNA Kit (BIOTOOLS Co., Ltd., Taipei, Taiwan.) as previously described [17]. cDNA was synthesized using a ToolScript MMLV RT kit (BIOTOOLS Co., Ltd., Madrid, Spain). q-PCR was carried out using a StepOnePlusTM system (Applied Biosystems, Foster City, CA, USA) with TOOLS 2X SYBR qPCR Mix (BIOTOOLS Co., Ltd.). The expression levels of all the genes in cells were normalized to the internal control *RNU6-1* gene. All the samples with a coefficient of variation for Ct value > 1% were retested.

### 2.6. Multi-Omics Analysis

The bioinformatics analysis follows the previous publication [18,19] and is briefly described as follows:

Mutations and co-expression of *ACSS2* were calculated and analyzed using the CBio Cancer Genomics Portal (cBioPortal) database. The search term “*ACSS2*” was used to obtain information on mutation distribution and patient genetic variants for all tumor and non-tumor tissues [20].

GEPIA2 (Gene Expression Profiling Interactive Analysis 2) is a Web-based tool and database that provides fast and customizable functionality based on TCGA and GTEx data. GEPIA 2 provides key interactivities including differential expression analysis, correlation analysis, patient survival analysis, similar gene detection, and downscaling analysis [20].

Protein–protein interaction (PPI) was confirmed by comparing the pathway of another species with its homolog from the Reactome and STRING interaction network and using protein-compound interactions from external databases for possible pathways [21,22,23].

The association between gene expression and immune cell infiltration/abundance in the TCGA dataset was explored through the “gene” module of TIMER. CD8+ T cells and cervical cancer-associated fibroblasts (CAF) were selected for analysis in this study. Immune infiltration levels were estimated by algorithms including TIMER, EPIC, MCPCOUNTER, CIBERSORT, CIBERSORT-ABS, QUANTISEQ, and XCELL. The relevant results are shown as heatmaps.

### 2.7. Immunofluorescence

The tissue was fixed with 4% paraformaldehyde for 10 min at RT, permeabilized with 0.2% Triton X-100 for 5 min, blocked with 5% BSA for 0.5 h, and incubated with the indicated 1st antibodies overnight at 4 °C. After washing with PBST, the samples were incubated with the secondary antibodies for 0.5 h at RT. All glass slides were digitized with BX61VS^®^ Fully Motorized Fluorescence Microscope (Olympus Corporation, Tokyo, Japan) at ×20 (0.26 μm/pixel) with High precision (High precision autofocus). BX61VS whole-slide images were viewed and analyzed with Olympus VS-ASW^®^ software at Li- Tzung Pathology Laboratory (Kaohsiung, Taiwan).

### 2.8. Statistical Analyses

Statistical methods were as previously described [17]. Correlation of gene expression was assessed using Spearman’s correlation coefficient. Statistical differences were analyzed using GraphPad Prism 8.0 (GraphPad Software, La Jolla, CA, USA) by performing a *t*-test or Fisher’s exact test for both groups and a one-way ANOVA test for one group. Kaplan–Meier curves were plotted to investigate survival trends, and *p*-values were evaluated using a log-rank test. During the experiment, the researchers turned a blind eye to group assignment. A *p*-value of less than 0.05 was considered statistically significant. Statistical significance, * *p* value < 0.05; ** *p* value < 0.01; *** *p* value < 0.001.

## 3. Results

### 3.1. Types of Cervical Cancer, Mutation Load, and Copy-Number Alterations

**Figure 1:**
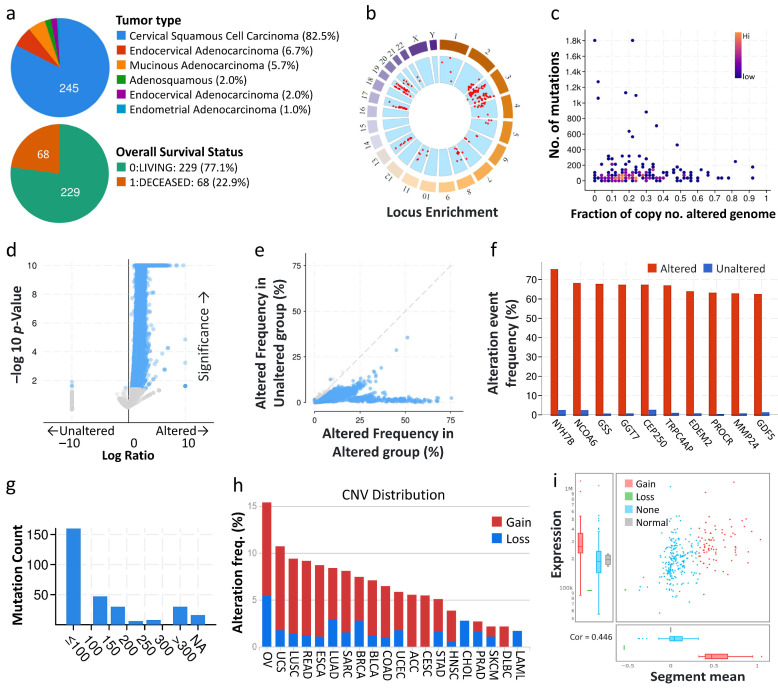
Analysis of global genes associated with mutations in *ACSS2*. (**a**) The percentage of each Cervical cancer type and overall survival in the TCGA dataset from cBioportal. (**b**) The circle plot provides a combination of scatter plot and box plot to show the CNV distribution and correlation among cervical cancer types. (**c**) Mutation counts versus fraction of genomes altered by copy-number changes for different kinds of CESC in the TCGA dataset. Volcano (**d**) and scatter plots (**e**) exhibiting genes associated with mutations altered frequency in *ACSS2*. (**f**) Box blot representing the 10 most frequently altered genes. (**g**) Mutation counts in patients with different kinds of cervical cancer in the TCGA dataset. (**h**) A pan-cancer global view of copy number variation (CNV) features based on *ACSS2* with increased gene expression potentially induced by copy number gains (CNGs). (**i**) The distribution and correlation of CNV in cervical cancer were marked with red (gain) and green (loss) to visualize the distribution of log_2_ ratios.

To determine the most prevalent type of gynecologic malignancy (cervical), we analyzed the TCGA dataset for cervical cancer (*n* = 607). The majority of cervical cancer malignancies were attributed to cervical squamous cell carcinoma (82.5%) and endocervical adenocarcinoma (6.7%), mucinous adenocarcinoma (5.7%), and endometrial adenocarcinoma (1.0%) occurred at lower frequencies. The overall survival rate was 229 cases (77.1%) survived and 68 cases (22.9%) died (Figure 1a). The “Locus Enrichment” shows the locus of all genes in all chromosomes. Each red dot represents a gene and its associated position on the chromosome. Each gene has a correlation between RNA expression and CNV (Figure 1b). To determine the mutation burden due to copy number alterations, we plotted the ratio of copy number to cancer genomes with copy number alterations for each cancer type. Patients with cervical cancer had a relatively low number of mutations but a relatively high number of copy number changes (Figure 1c). In addition, altered ACSS2 mutation (missense mutation, truncating mutation and fusion) frequency had few associations with that in other genes (Figure 1d,e). The 10 most frequently altered genes were MYH7B, NCOA6, GSS, GGT7, CEP250, TRPC4AP, EDEM2, MMP24, and GDF5 (Figure 1f). Cancer-specific mutations play an important role in prognosis and can be used as biomarkers to predict patient response to immunotherapy or chemoradiotherapy. Therefore, we assessed the mutation burden of each type of cervical malignancy by counting the mutations in each tumor sample. The majority of uterine cancer samples have less than 50 mutations. The majority of cervical tumors had a mutation burden in the <100 change range (Figure 1g).

**Figure 2:**
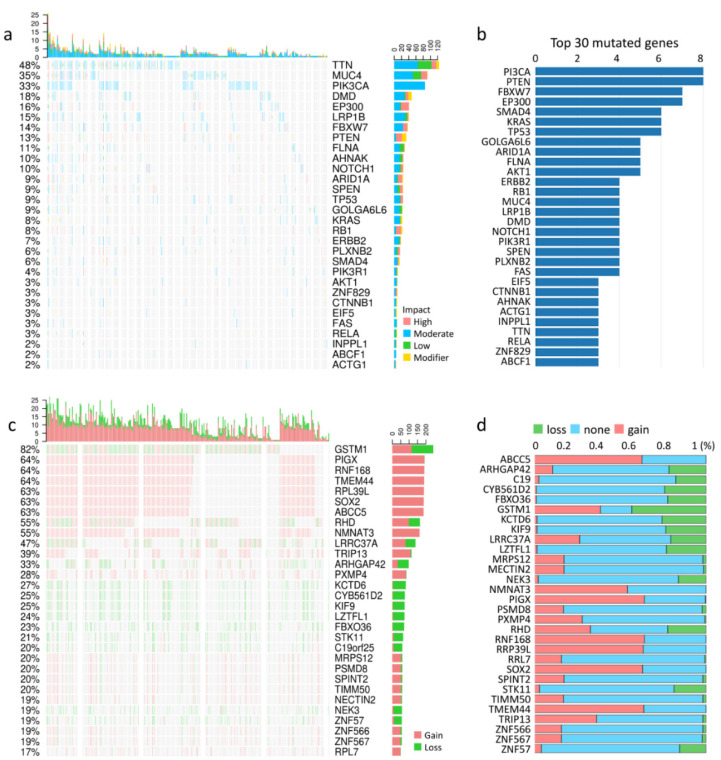
The landscape of mutation genes in CESC samples. (**a**) Waterfall Plot of the top 30 mutated genes from TCGA. The bar plot indicates the number of genetic mutations per patient, while the right bar plot displays the number of genetic mutations per gene. (**b**) The graph shows the top 30 genes on the *y*-axis and the number of mutations that define them on the *x*-axis. (**c**) Waterfall plot illustrates the relations between the top 30 genes and the CNV in cancer patients for a specific CESC cancer type. (**d**) The bar chart outlines the percentage of CNV for each of the top 30 genes in cervical cancer.

Tumor samples with the highest amplification frequency and the most significant overall survival values were selected for each cancer type. *ACSS2* accounted for 5.6% of the CESC amplification frequency and no loss frequency (Figure 1h,i). We also obtained the mutation profiles of CESC patients from the TCGA database to present the results (Figure 2a,b). In addition, we observed that *TTN* (48%), *MUC4* (35%), *PIK3CA* (33%), *DMD* (18%), *EP300* (16%), *LRP1B* (15%), *FBXW7* (14%), *PTEN* (13%), *FLNA* (11%) and *AHNAK* (10%) were the top 10 mutated genes in CESC (Figure 2a). We also summarize the concordant and exclusive relationships between mutated genes in Figure 2b, and use Genecloud plots to show the frequency of mutations in other genes. We further downloaded the mutation data of CESC samples from TCGA, and the cumulative mutations frequency in each gene was counted and sorted in decreasing order (Figure 2c,d). The top 30 frequently mutated genes with high mutation frequency and the pattern of somatic mutation for the top 30 genes are illustrated in Figure 2c. The top 10 genes and the CNV were *GSTM1* (82%), *PIGX* (64%), *RNF168* (64%), *TMEM44* (64%), *RPL39L* (63%), *SOX2* (63%), *ABCC5* (55%), *RHD* (55%), *NMNAT3* (47%), and *TRIP13* (39%). To conclude, the results suggested that ACSS2 mutations showed close associations with the tumorigenesis and prognosis of CESC.

### 3.2. Prognostic Value of Hub Genes

**Figure 3:**
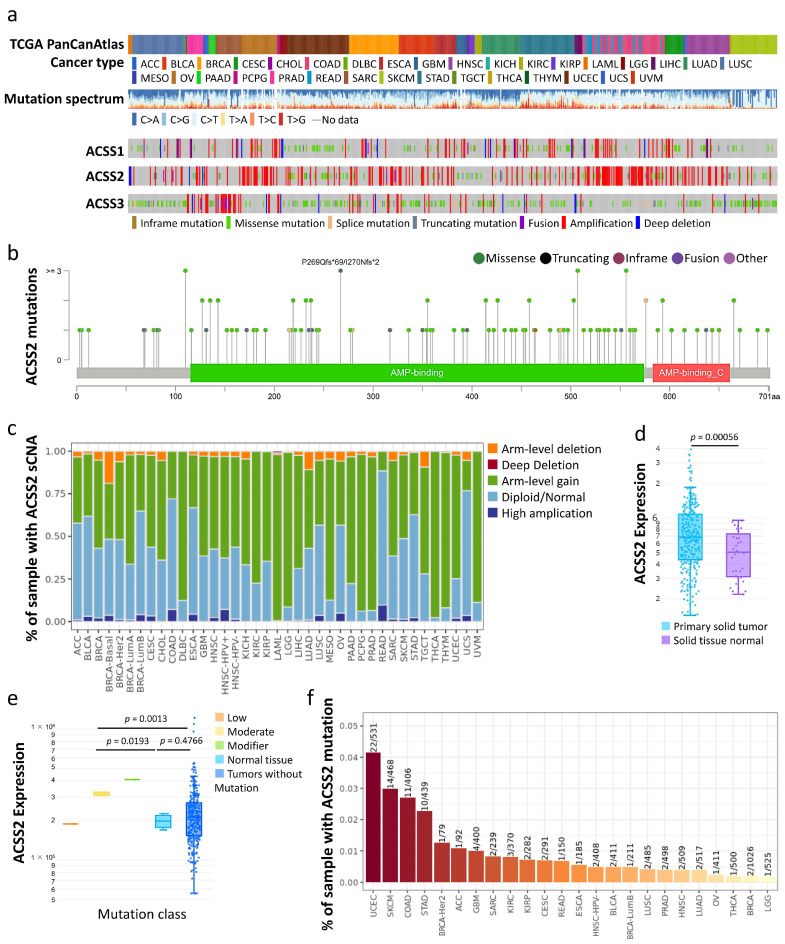
(**a**) Analysis of CNV mutations in ACSS-related genes in human cancer data of cBioportal platform. (**b**) The graphical view shows the ACSS2 protein domains and the positions of specific mutations. The length of the line connecting the mutation annotation to the protein is indicative of the number of samples that have the mutation. (**c**) An illustration on the definition of focal, arm, and chromosome level somatic copy-number alterations (sCNA) of ACSS2. (**d**) ACSS2 expression in CESC tumor tissues compared to non-tumor tissues. (**e**) The expression of ACSS2 in different types of mutant CECS tumor tissues. (**f**) The mutation module analyzes and visualizes the influence of gene mutations on immune cell infiltration in a variety of cancer types and immune cell types.

To understand the genetic changes in the ACSS family, we found that 2.4% of the *ACSS2* gene was mutated in various cancers by querying the copy number change data and mutation percentage of various tumor samples recorded in cBioportal (Figure 3a). From the diagram of *ACSS2* gene and the encoded protein, mutations occurred more frequently in the AMP-binding domain that is responsible for heteromerization and transactivation (Figure 3b). To investigate the relationship between SCNA and ACSS2, we first examined the expression of a number of representative genes from each of the major ACSS2 pathways. We observed the gene expression levels of ACSS2 master regulators in each tumor (Figure 3c). From the TIMER database, we found a significant increase in *ACSS2* expression in tumor tissues compared to normal controls in CESC (Figure 3d). Next, we investigated the relationship between ACSS2 expression and CESC mutation type. The results showed significant differences between the moderate and normal tissue and tumors groups (Figure 3e). We analyzed the effect of *ACCS2* mutations on immune cell infiltration in various cancer types and immune cell types by mutation module in pan-cancer. We found that mutations in ACCS2 affect the immune response (Figure 3f).

**Figure 4:**
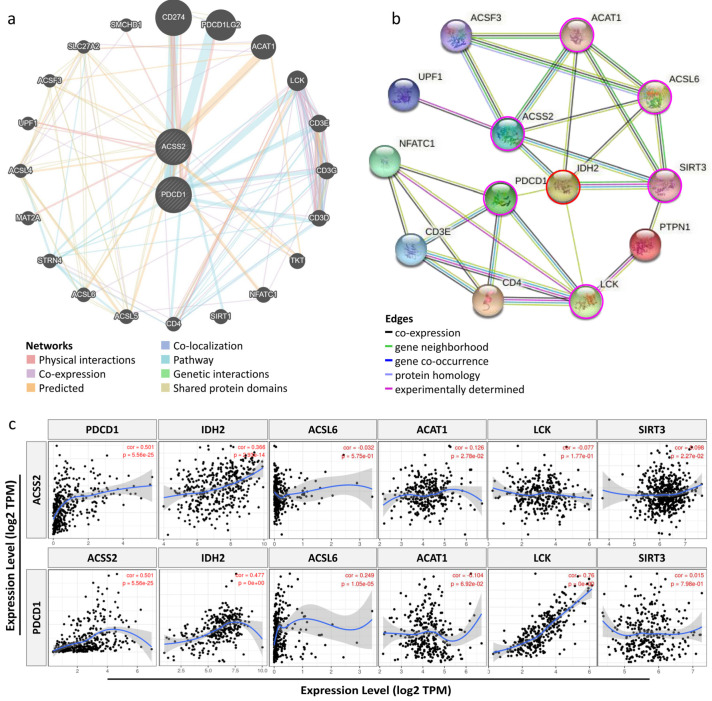
Identifying the interactive network of ACSS2 and the PPI network with similar functions. (**a**) An interaction network for ACSS2 was generated through the Reactome database. The functionally similar genes were located in the outer circle, while hub genes were located in the inner circle. The color of nodes was related to the protein function while line color represented the type of protein interaction. (**b**) A PPI network for ACSS2 was generated through the STRING database. (**c**) Scatterplots showing the correlation between ACSS2, PDCD1, IDH2, ACSL6, ACAT1, LCK, and SIRT3 expression in CESC.

To further confirm the association of ACCS2 with immunity. We constructed a gene–gene interaction network of *ACSS2* and *PDCD1*. The middle node represents *ACSS2* and *PDCD1*, and the surrounding 20 nodes represent genes associated with ACSS2. Functional analysis showed that these proteins were significantly associated with energy metabolism and immune response (Figure 4a). To further investigate the biology of ACSS2 and PDL1, a PPI network containing 10 nodes was generated through the STRING online database (Figure 4b). The five most important nodes were IDH2, ACSL1, ACSL6, LCK, and SIRT3. Therefore, to investigate whether ACSS2 expression in CESC also increases immunosuppressive cell-derived cytokines, we analyzed the correlation between ACSS2 expression and cytokine gene markers (IDH2, ACSL6, ACAT1, LCK, SIRT3) using the TIMER database. As shown in Figure 4c, ACSS2 expression was significantly correlated with gene expression of immunosuppressive cytokines in CESC. However, PDCD1 expression in CESC was correlated with gene expression of immunosuppressive cytokines compared to ACSS2.

### 3.3. Association of the ACSS2 and PDL1 Levels with Cervical Tumorigenesis and Clinicopathological Outcomes

**Figure 5:**
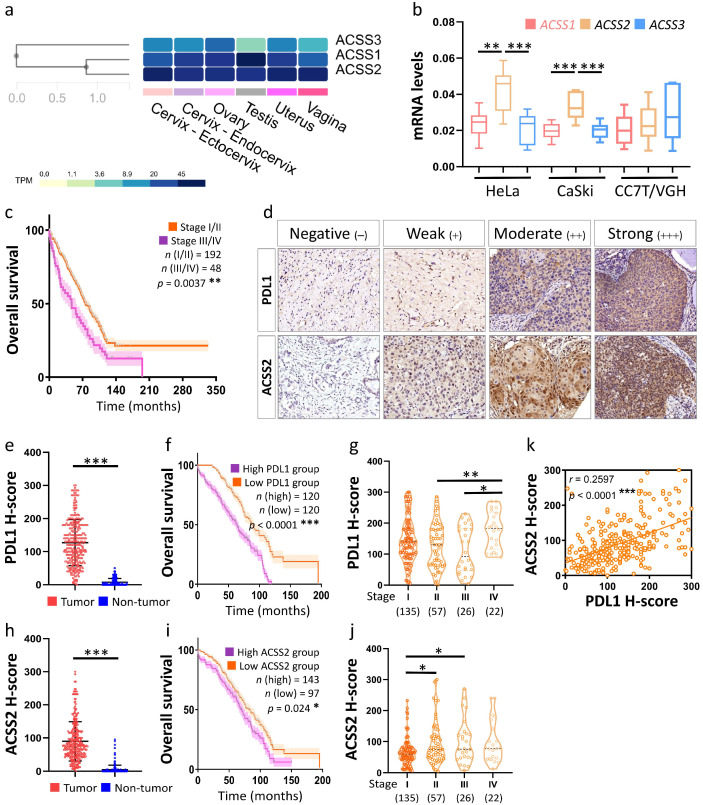
Diagnostic value of PDL1 and ACSS2 expression in tumor biopsies during CESC occurrence. (**a**) Heat map representing the ACSS family index of all tissues in the Web-based Gene Set Analysis Toolkit. (**b**) qRT-PCR was used to detect the expression levels of different cervical cancer cells and RNU6-1 was used as an internal control. (**c**) Staging and survival of clinical patient specimens were analyzed. (**d**) Comparison of representative micrographs of PDL1 and ACSS2 between normal and CESC tissues. Representative images of PDL1 and ACSS2 negative, weak (+), moderate (++) and strong (+++) staining in CESC tissues. (**e**,**h**) IHC scores of PDL1 and ACSS2 expression in CESC tissues and matched normal tissues. (**f**,**i**) Kaplan–Meier survival curves for disease-specific survival according to PDL1 and ACSS2 expression status. (**g**,**j**) Violin plots of gene expression levels in CESC with significant alterations in different stages. (**k**) Scatter plot illustrating the Spearman’s correlation of normalized reads per patient between ACSS2 and PDL1. * *p* < 0.05, ** *p* < 0.01, *** *p* < 0.001.

To confirm the importance of ACSS2 in cervical cancer, we compared the levels of *ACSS1~3* in the TPM of the female genital tract and the results showed that *ACSS2* was abundant in the cervix (Figure 5a). We further tested the mRNA levels of *ACSS1~3* in multiple cervical cancer cells and our results revealed that the expression of *ACSS2* was higher than the other two genes in Hela and CaSki cells (Figure 5b). We diagnosed and collected specimens of cervical cancer patients and benign patients from Kaohsiung Veterans General Hospital. Statistical analysis revealed that the cancer stage was correlated with patient survival (Figure 5c). After excluding cases with incomplete pathological and clinical data, TMA analysis was performed on 240 cervical cancer patient samples (Figure 5d). For each patient sample block, two tumor tissue spots and one normal control spot were used for TMA; IHC analysis of ACSS2 and PDL1 was used as an example. The IHC staining intensity of ACSS2 and PDL1 was scored as negative = 0, weak = 1, moderate = 2 or strong = 3, and this score was multiplied by the percentage of positive IHC staining cells to obtain a histological score (H-score) for further analysis. The H-score of TMA samples showed that the expression of PDL1 and ACSS2 was significantly higher in cervical tumor (T) tissues than in non-tumor (N) tissues (Figure 5e,h). Similar to the above TCGA data, the overall survival rate of CESC patients with high PDL1 and ACSS2 mRNA expression was lower than that of CESC patients with low PDL1 and ACSS2 mRNA expression (Figure 5f,i). In addition, both genes showed similar trends in different stages, with a clear trend of increasing PDL1 scores in stage IV and stage II–III, and an increasing trend of ACSS2 scores in stage I and stage II–III (Figure 5g,j). We further analyzed the H-score of 240 patients for correlation and showed a positive correlation between ACSS2 and PDL1 expression (Figure 5k).

**Figure 6:**
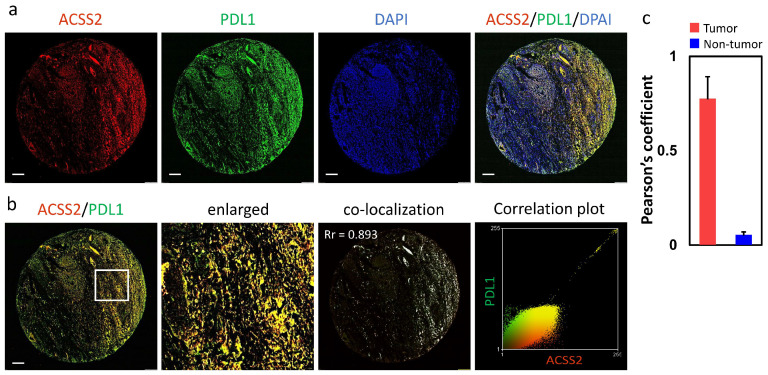
Colocalization analysis of ACSS2 with PDL1 in tumor biopsies during CESC occurrence. (**a**) The representative images of CESC stained with ACSS2, PDL1, and DAPI. The enlarged images in (**b**) highlight the representative co-localization with 20× magnification from white squares in the overlay images. Co-localization analyses of ACSS2 and PDL1. The co-localization in (**c**) is presented as the product of the differences from the mean image. White color pixels indicate co-localization coefficient. Scale bar: 200 µm.

To confirm the association of ACSS2 with PDL1 in the CESC, the co-localization of these two proteins in the TMA was investigated. The whole CESC was triple-labeled by immunofluorescence and labeled with ACSS2, PDL1, and DAPI. Similar to the results in Figure 5k, the fluorescence intensity of ACSS2 and PDL1 increased significantly with malignant tumors (Figure 6a). In Figure 6b, the red fluorescence ACSS2 and the green fluorescence PDL1 are shown, while the overlap of the two fluorescences is shown as yellow, and the overlap ratio of the two fluorescences is further analyzed by Pearson, which indicates that ACSS2 co-localizes with PDL1. In addition, the overall co-localization of the full field of view was calculated and the Pearson coefficient of tumor versus non-tumor was observed from 0.776 versus 0.055 compared to non-tumor (Figure 6c).

### 3.4. Correlation Analysis between ACSS2 Expression and Major Types of Cancer Immune Response

**Figure 7:**
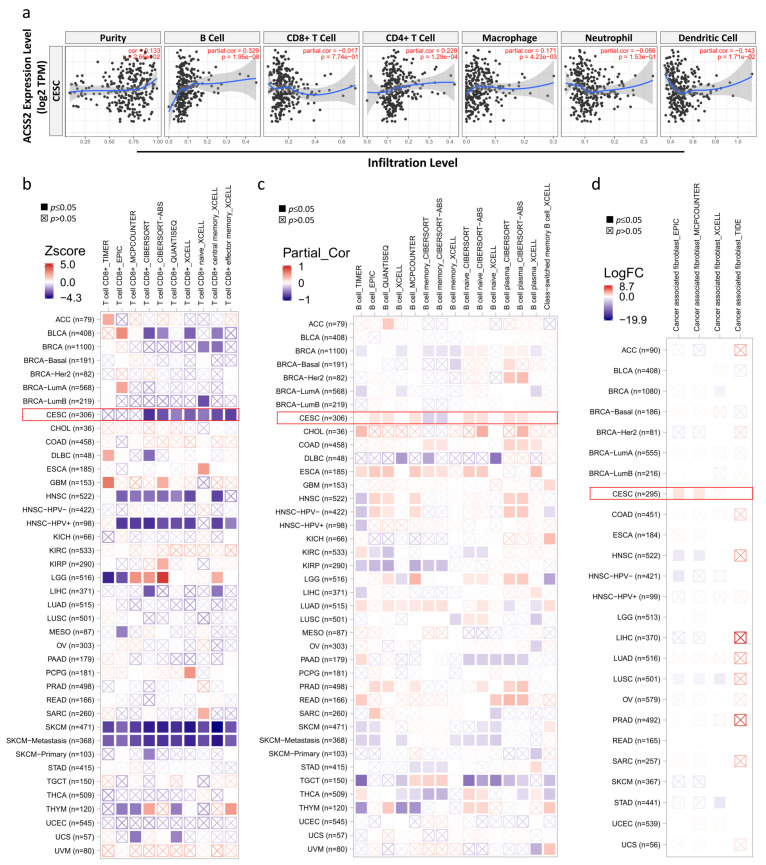
Association of ACSS2 expression with tumor immune microenvironment factors. (**a**) TIMER database analysis shows correlation between ACSS2 expression levels in CESC patient tissues and tumor infiltration levels of immune cell types, namely, B cells, CD8+ T cells, CD4+T cells, macrophages, neutrophils, and dendritic cells. (**b**) Correlation between ACSS2 expression and immune infiltration of T cells, (**c**) B cells, (**d**) and cancer-associated fibroblasts (CAFs) across different cancers in TCGA dataset.

We evaluated the relationship between ACSS2 and diverse tumor-infiltrating immune cells. The results from the TIMER database suggested that ACSS2 was remarkably correlated not only with tumor purity but also with the infiltrating levels of different immune cells, including CD4+ T cells, CD8+ T cells, B cells, neutrophils, macrophages, and dendritic cells, in CESC (Figure 7a). These results suggest that *ACSS2* is tightly connected with the infiltration of immune cells in cervical cancer, especially the infiltration of macrophages. The immune microenvironment plays a key role in both tumor progression and elimination; therefore, it is interesting to analyze the association between *ACSS2* expression and pro-/anti-tumor immune components. We used seven algorithms to quantify the density of CD8+ T cells in each cancer type and then correlate it with *ACSS2* expression levels. We observed an overall negative correlation between CD8+ T-cell fraction and *ACSS2* expression in pan-cancer, except for GBM, where both components were negatively correlated based on all algorithms (Figure 7b). The difference is that there is a positive correlation between B cell scores and *ACSS2* expression (Figure 7c). Our analysis showed that *ACSS2* expression and CAF abundance were negatively correlated in most of the cancer types (Figure 7d). Notably, based on three of the four algorithms (EPIC, MCPCOUNTER, TIDE, and XCELL), a significant positive correlation between *ACSS2* expression and CAF was observed in CESC (Figure 7d, Table 1).

### 3.5. Relationship between ACSS2 and Distinct Immune Markers Sets

**Figure 8:**
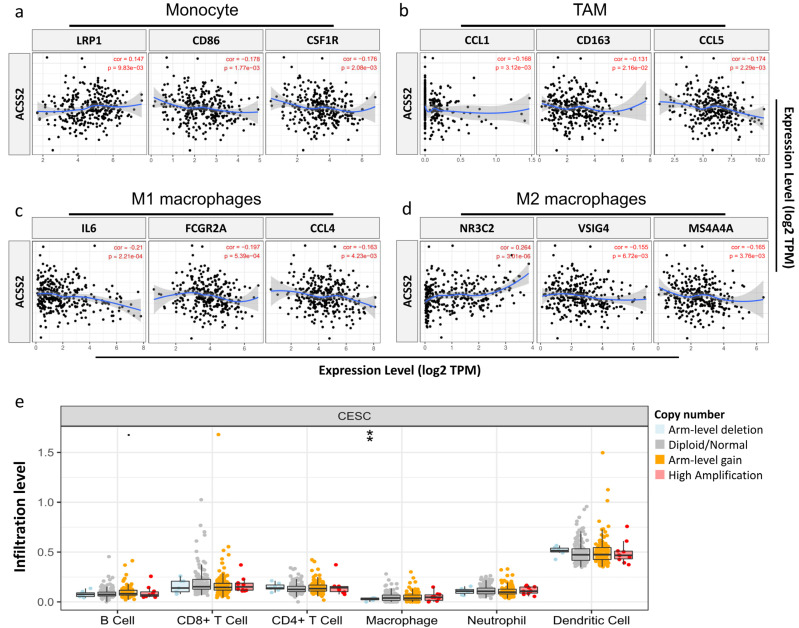
Correlations between the CNV of ACSS2, immune cell infiltration, and prognosis in CESC. Relationship between ACCS2 and various gene markers of (**a**) monocytes, (**b**) TAMs, (**c**) M1 macrophages, and (**d**) M2 macrophages in CESC. (**e**) High amplification of ACSS2 in macrophage cells in CESC. ** *p* < 0.001. TAM: Tumor-associated macrophages.

ACSS2 expression was significantly correlated with the levels of most markers in different types of immune cells in CESC. Because macrophages are the immune cell type most closely associated with ACSS2 expression (Figure 8a–d), we further investigated the association between ACSS2 and the immune marker set of monocytes, tumor-associated macrophages (TAM), M1 macrophages, and M2 macrophages. In CESC tissues, *ACSS2* was positively associated with TAM infiltration, but not in the corresponding normal cervical tissues. In addition, we further investigated the association between CNV of IRGs in the prognostic model and immune cell infiltration using the TIMER Web server. The results showed that the absence or expansion of other forms of copy number may differentially inhibit the infiltration of immune cells compared to normal copy number (Figure 8e). In addition, a high level of B cells suggested good prognosis of CESC, and high expression of ACSS2 may prompt better survival.

## 4. Discussion

Metabolic variation is an important factor in the progression of cancer. Due to the rapid demand for energy, cancer cells drive a reprogramming of the glucose metabolism mechanism, which increases the proportion of glucose glycolytic metabolic pathways, with only a small proportion going through the mitochondrial aerobic respiratory pathway. This phenomenon, known as the Warburg effect, is present in almost all tumor cells, and cancer cells are also addicted to this rapid energy-generating glycolytic pathway [24].

Warburg reported in 1927 that tumor fermentation (glycolysis) required about two-thirds of the blood glucose. These findings were initially interpreted as indicating that mitochondria had become defective and therefore cancer cells were dependent on increased glycolysis for ATP production. Other metabolic changes included increased glutaminolytic metabolism [24] and increased lactate production [25]. However, these metabolic changes are typical of non-transforming, rapidly dividing cells, which tend to abandon ATP production through oxidative phosphorylation, favoring rapid glucose turnover and increased pentose–phosphate flux, which leads to lactate production. How are these changes faithfully manifested in cancer cells and how are they maintained by a repetitive cell cycle? The control of mitochondrial intermediate metabolism is partially regulated by acetylation [26]. Similarly, acetylation of histone and non-histone proteins in the nucleus is a key factor in the epigenetic regulation of gene transcription through selective chromatin remodeling and transcription factor activity. Protein acetyltransferases (KAT) and deacetylases (KDAC) act as key regulators of these processes. Sirtuins are NAD^+^-dependent protein deacetylases that also regulate cellular behavior through selective deacetylation, including the deacetylation of ACSS1 and ACSS2 [27].

Previous studies have identified multiple roles for metabolites in the regulation of immune responses. Thus, metabolites provide substrates for biosynthetic reactions that support proliferative responses during T-cell expansion, for example [28,29]. Metabolites can influence transcriptional regulation in the innate and adaptive immune systems through epigenetic mechanisms, mainly through post-translational modifications of histones [30,31,32,33]. The most studied histone modifications are histone methylation and acetylation. Histone methylation is associated with transcriptional activation and inhibition, depending on the histone residues involved. It is affected by the tricarboxylic acid (TCA) cycle metabolites ketoglutarate, succinate, and fumarate, which provide precursors for the methyl donor S-adenosylmethionine by affecting histone demethylase activity, and by the amino acid methionine [34,35,36]. Histone acetylation is associated with transcriptional activation, leading to chromatin opening [37]. It can also be achieved by metabolic control mainly through the acetyl donor acetyl coenzyme A [38].

Several studies have shown that immune cells are in an abnormally activated state when they become exhausted phenotypically due to the elimination of tumor cells, which can not only upregulate the expression of immunosuppressive cytokines but can also directly lead to immunosuppression [39]. This can not only upregulate the expression of immunosuppressive cytokines but also directly lead to immunosuppression [40]. In response to chronic stimulation by tumor antigens, exhausted CAF continue to activate the expression of ACSS2 and other immune checkpoint receptors, which further promotes tumor invasion. Here, we confirmed the upregulation of *ACSS2* in CESC tissues through data mining and in vitro experiments and revealed that *ACSS2* is associated with poor prognosis. In contrast to other studies, this study confirmed that ACSS2 plays an important role in the regulation of T cells and represents a greater infiltration of TIL into TME, especially B cells and CAF. However, in the high ACSS2 group, the phenotype of TME tended to be immunosuppressive, while the infiltrated CAF tended to be exhausted, synergizing with Tregs and ultimately leading to tumor metastasis and progression.

This study has some limitations. First, only computational, in vitro experiments and patient tests were performed. Further confirmation using specific animal models is required. Next, only 240 CESC patients were available for TMA analysis and biopsies should be collected continuously in the future to confirm the association between ACSS2 and PDL1 at different times. Genetic mutations, epigenetic, and proteomic differences should also be considered in future studies. Finally, despite the multi-component validation of this study, the molecular mechanisms of the tumor microenvironment still need to be validated and further confirmation through multi-center is required.

## 5. Conclusions

In conclusion, this study provides new insights into the potential role of ACCS2 in oncologic immunology and its prognostic value. ACCS2 levels correlated with the prognostic and immune infiltration levels of CESC, suggesting that it could be used as a prognostic biomarker. Therefore, the development of ACCS2 inhibitors would have the potential to interfere with immune cells to achieve therapeutic strategies.

## Figures and Tables

**Table 1:** The relationship of clinicopathological characteristics with ACSS2 and PDL1 expression in CESC patients.

		ACSS2 Expression			PDL1 Expression		
Variables	Total	Low	High	*p*-Value	Low	High	*p*-Value
Case number, n	240	97	143	-	120	120	-
Age	55.0 ± 13.5	53.4 ± 13.2	55.7 ± 13.7	0.36	54.1 ± 13.8	56.3 ± 13.1	0.42
Survive, n	240	143	97	0.02	120	120	<0.001
Recurrence, n	194	72	122	0.39	108	86	0.43
FIGO stage, n							
I	135	62	73	0.04	94	41	<0.01
II	57	29	28	-	27	30	-
III	26	10	16	-	5	21	-
IV	22	10	12	-	6	16	-
Differentiation, n							
Well	77	41	36	0.03	11	66	<0.01
Moderate/Poor	163	61	102	-	32	131	-

## Data Availability

Links to publicly archived datasets analyzed in this study have been provided in the “Material and Methods” section.

## Abbreviations

ACC: Adrenocortical carcinoma, BLCA: Bladder urothelial carcinoma, BRCA: Breast invasive carcinoma, CESC: Cervical and endocervical cancers, CHOL: Cholangiocarcinoma, COAD: Colon adenocarcinoma, DLBC: Lymphoid Neoplasm Diffuse Large B-cell Lymphoma, ESCA: Esophageal carcinoma, GBM: Glioblastoma multiforme, HNSC: Head and Neck squamous cell carcinoma, KICH: Kidney Chromophobe, KIRC: Kidney renal clear cell carcinoma, KIRP: Kidney renal papillary cell carcinoma, LAML: Acute Myeloid Leukemia, LGG: Brain Lower Grade Glioma, LIHC: Liver hepatocellular carcinoma, LUAD: Lung adenocarcinoma, LUSC: Lung squamous cell carcinoma, MESO: Mesothelioma, OV: Ovarian serous cystadenocarcinoma, PAAD: Pancreatic adenocarcinoma, PCPG: Pheochromocytoma and Paraganglioma, PRAD: Prostate adenocarcinoma, READ: Rectum adenocarcinoma, SARC: Sarcoma, SKCM: Skin Cutaneous Melanoma, STAD: Stomach adenocarcinoma, TGCT: Testicular Germ Cell Tumors, THCA: Thyroid carcinoma, THYM: Thymoma, UCEC: Uterine Corpus Endometrial Carcinoma, UCS: Uterine Carcinosarcoma, UVM: Uveal Melanoma.
